# The complete plastid genome of cherry plants *Prunus sargentii* (Rosaceae) and its phylogenetic implication

**DOI:** 10.1080/23802359.2021.1935355

**Published:** 2021-08-19

**Authors:** Yan-Feng Song, Qi Ye, Meng Li, Jie Chen, Xian-Gui Yi, Xian-Rong Wang, Shao-Jun Wang

**Affiliations:** a Co-Innovation Center for Sustainable Forestry in Southern China, College of Biology and the Environment, Nanjing Forestry University, Nanjing, PR China; b Meishan Shengshi Landscaping Co., Ltd, Meishan, PR China

**Keywords:** Chloroplast genome, *Prunus*, Cerasus

## Abstract

*Prunus sargentii* is an ornamental flowering cherry species, spread in Japan, Korea, Russia, and Northeast China. Little information is available regarding its genomic, with limited phylogenetic relationship study performed on *P. sargentii* until now. In this research, we reported the complete plastid genome of *P. sargentii.* The complete chloroplast of this species is 158,138 bp in length, including a pair of invert repeat regions (IR) (26,463bp) that is divided by a large single-copy region (LSC) (85,959bp) and a small single-copy region (SSC) (19,253bp). The plastid genome contained a total of 128 genes, including 84 coding genes, eight *rRNA* genes, and 36 *tRNA* genes. Phylogenetic analysis indicates that *P. sargentii* has a closer relationship with *P. kumanoensis*.

*Prunus* L. subg. *Cerasus* (Mill.) A. Gray contains approximately 150 species that mainly occupy temperate and subtropical regions of the northern hemisphere (Yu et al. 1986). Since subg. *Cerasus* provides various edible cherries and ornamentals of economic value, these taxa have great potential for development and application (Li et al. 2019). *Prunus sargentii* Rehder (Schwerin and Beissner 1908, p. 159), commonly called Sargent cherry or North Japanese hill cherry, is a kind of graceful ornamental flowering cherry trees. This species is mainly distributed in Japan, Korea, Russia, and Northeast China. Meanwhile, the genetic relationship of *P. sargentii* relative to other subg. *Cerasus* is poorly understood. Therefore, we sequenced the whole chloroplast genome of *P. sargentii* to elucidate its phylogenetic relationship with other subg. *Cerasus* species.

The plant material was obtained from Dongling, Liaoning province, China (41°83′65″N 123°58′34″E, altitude 90 m). A specimen was deposited at Nanjing Forestry University (https://shengwu.njfu.edu.cn/; collector: Meng Li, limeng@njfu.edu.cn; voucher number: NF: 161098816). Total DNA was extracted from fresh leaves with a modified CTAB protocol. The whole-genome sequencing was conducted by Nanjing Genepioneer Biotechnologies Inc. (Nanjing, China) on the Illumina Hiseq 2500 platform (Illumina, San Diego, CA). A total of 3.54 Gb clean PE reads (Phred scores >20) were assembled using the program GetOrganelle version 1.7.2 (Jin et al. 2020). The plastome was annotated by the web application GeSeq (https://chlorobox.mpimp-golm.mpg.de/geseq.html) (Tillich et al. 2017).

The complete circular plastid genome of *P. sargentii* (GenBank accession No. MW392082) was 158,138 bp in length. Consisting of four regions; large single-copy region (LSC) of 85,959 bp, small single-copy region (SSC) of 19,253 bp, and a pair of inverted repeat regions (IRA and IRB) of 26,463 bp each. The overall GC contents of the plastid genome were 36.7%; LSC (34.6%), SSC (30.3%), and IR (42.5%). The genome contained a total of 128 genes, including 84 coding genes, eight *rRNA* genes, and 36 *tRNA* genes.

Phylogenetic analysis including *P. sargentii*, 20 other subg. *Cerasus* species and two outgroups of subg. *Prunus* were performed using complete plastid genomes. Sequences were aligned by MAFFT version 7.467 (Katoh et al. 2005) and visually checked and adjusted in Bioedit. Maximum-likelihood (ML) analysis was conducted in IQ-TREE version 2.1.1 (Vergara et al. 2015). The result was well-resolved and revealed that *P. sargentii* was belonged to subg. *Cerasus* and most closely related to *P. kumanoensis* (Figure 1). In summary, the complete plastid genome of *P. sargentii* will provide useful genetic information for increasing the richness of subg. *Cerasus*, as well as assisting in phylogenetic and evolutionary studies of subg. *Cerasus*.

**Figure 1.**
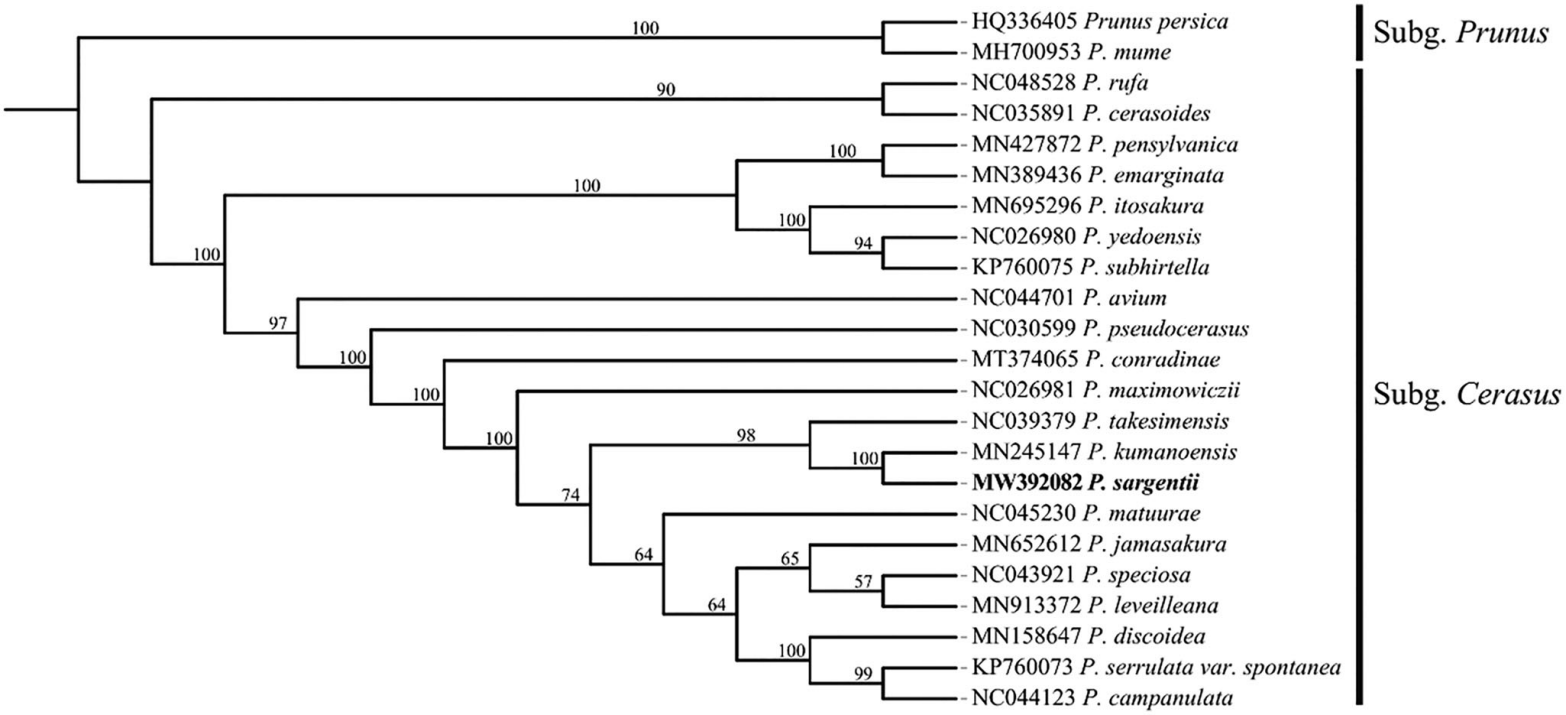
Maximum-likelihood phylogenetic tree for *P. sargentii* based on 23 complete plastid genomes. *P. persica* and *P. mume* (Rosaceae) were used as outgroup and the support values are displayed above the branches.

## Data Availability

The plastid genome in this study is available in the NCBI GenBank (https://www.ncbi.nlm.nih.gov/genbank/) with an accession number MW392082. The associated BioProject, SRA, and Bio-Sample numbers are PRJNA686839, SRR13279544, and SAMN17126435, respectively.
